# Risk factors associated with hearing loss in neonates: A retrospective cross-sectional study from Qatar

**DOI:** 10.5339/qmj.2025.99

**Published:** 2025-12-01

**Authors:** Jarreth Noël Andreas, Woldekidan Kifle Amde, Rifqah Abeeda Roomaney

**Affiliations:** Division of Pediatric Otolaryngology and Audiology, Sidra Medicine, Ar Rayyan, Doha, Qatar; School of Public Health, University of the Western Cape, Bellville, Cape Town, South Africa; Burden of Disease Research Unit, South African Medical Research Council, Francie van Zijl Drive, Parow Valley, Cape Town, South Africa *Email: Jarreth Noël Andreas jandreas@sidra.org

**Keywords:** Hearing loss, neonatology, risk factors, Qatar, audiology, public health

## Abstract

**Background:**

Hearing loss (HL) affects approximately 3 in every 1,000 infants worldwide. However, current Early Hearing Detection and Intervention (EHDI) guidelines may not fully account for region-specific neonatal risk factors. In the Middle East, conditions such as gestational diabetes mellitus (GDM), pre-eclampsia, and consanguinity are highly prevalent but remain underexplored in HL research. This study investigates the association between these and other neonatal risk factors and HL outcomes in newborns in Qatar.

**Methods:**

A retrospective cross-sectional review of electronic health records was conducted for 5,098 neonates born between March 2019 and March 2022 at a tertiary hospital in Qatar. After exclusions, 4,126 neonates were analyzed. Maternal and neonatal risk factors, along with newborn hearing screening outcomes, were assessed using Fisher’s exact test, Chi-square test, and logistic regression analysis.

**Results:**

Overall, 6% of neonates failed the hearing screening (*n* = 248). The most common risk factors were GDM (34.5%), hyperbilirubinemia (21.0%), and gentamicin exposure (9.6%). However, GDM showed no significant association with failed screening. The strongest association was found with Down syndrome (odds ratio [OR] = 14.215; 95% confidence interval [CI]: 4.286–47.151; *p* < 0.001), followed by cleft lip/palate (OR = 4.371; 95% CI: 1.384–13.801; *p* < 0.012), and high-risk categorization (OR = 1.973; 95% CI: 1.266–3.076;*p* < 0.003).

**Conclusion:**

Contrary to expectations, GDM and hyperbilirubinemia were not associated with an increased risk of HL. In contrast, Down syndrome and cleft lip/palate showed significant associations with HL risk. These findings highlight the importance of incorporating regionally relevant risk factors—such as genetic syndromes and craniofacial anomalies—into local Early Hearing Detection and Intervention (EHDI) frameworks. Contextualizing hearing screening within the Gulf region may enhance early detection and optimize care pathways.

## 1. INTRODUCTION

Hearing loss (HL) is a major global public health concern, affecting over 1.5 billion individuals worldwide, with an estimated 3.1% residing in the Eastern Mediterranean Region.^1^ In neonates, HL occurs in approximately 2–4 per 1,000 live births, with significantly higher rates observed among those admitted to the neonatal intensive care unit (NICU).^2^ Sensorineural hearing loss (SNHL), the most common type of permanent childhood HL, is associated with significant delays in speech, language, cognitive, and psychosocial development.^3–5^

The Joint Committee on Infant Hearing (JCIH) established risk-based screening guidelines in 2000, followed by a comprehensive update in 2007 that identified specific neonatal risk factors. These include a family history of HL, NICU admission for more than five days, exposure to ototoxic agents, hyperbilirubinemia requiring exchange transfusion, associated congenital syndromes, bacterial or viral meningitis, and caregiver concerns.^6,7^ While international guidelines have guided the development of Early Hearing Detection and Intervention (EHDI) programs globally, their effective application requires adaptation to local contexts.

In Qatar, newborn hearing screening was first introduced in 2003 and is now implemented across both public and private sectors, following a structured protocol that includes otoacoustic emissions (OAE) and automated auditory brainstem response (AABR) testing at birth, 2 months, and 1 year of age.^8^ While universal newborn hearing screening has become standard practice, there remains a need to examine context-specific risk factors influenced by culture, tradition, and geographical location. Failure to consider region-specific risk factors may reduce the effectiveness of universal newborn hearing screening programs, resulting in delayed identification and intervention.

For instance, consanguinity, maternal gestational diabetes mellitus (GDM), and pre-eclampsia are highly prevalent in the Middle East and have been identified as potential risk factors for congenital HL.^9–11^ However, their inclusion in global hearing screening protocols, such as those outlined by the JCIH, remains limited. This highlights a potential gap in risk profiling, particularly in regions where these conditions are both endemic and culturally linked.

GDM affects multiple organ systems, and its impact during gestation is particularly concerning. Elevated levels of glucose, lipids, and amino acids in the maternal bloodstream create a suboptimal intrauterine environment that can adversely affect fetal development.^12^ These maternal metabolic disturbances may pose serious risks to the development of the neonatal auditory system.^12^

Similarly, hyperbilirubinemia (jaundice) is a common clinical condition affecting a significant proportion of newborns worldwide.^13^ Although the relationship between bilirubin levels and neurodevelopmental outcomes has been well explored, the findings remain inconclusive. Evidence suggests that elevated bilirubin levels may lead to auditory and neurological impairment, though individual susceptibility varies.^13–15^ From an audiological perspective, bilirubin neurotoxicity is believed to primarily affect the auditory brainstem nuclei—particularly the inferior colliculi and spiral ganglion neurons^14^—highlighting the need for early detection and intervention.

Calls have been made to either update the JCIH risk register or to develop context-specific guidelines, which are tailored to regional epidemiology and healthcare infrastructure.^5,16,17^ Despite its widespread adoption, the JCIH framework was primarily developed for the United States, and may not fully address the sociocultural and clinical risk profiles observed in low- and middle-income countries (LMICs) and Gulf states.

This study aims to contribute to the regional evidence base by examining the prevalence of HL-related risk factors and their association with failed newborn hearing screening outcomes among neonates born at a tertiary pediatric hospital in Qatar. To our knowledge, this is among the first studies in the Middle East to explore the predictive value of maternal conditions such as GDM and pre-eclampsia on neonatal hearing outcomes.

The objectives of this study were as follows:

1. To determine the proportion of neonates who failed the initial hearing screening between March 2019 and March 2022.
2. To identify the prevalence of selected maternal and neonatal risk factors within the study sample.
3. To investigate the strength of association between the identified risk factors and failed hearing screening outcomes.

## 2. METHODS

### 2.1. Study design, sample, and setting

This retrospective cross-sectional study was conducted at Sidra Medicine, a tertiary pediatric hospital in Qatar. De-identified electronic medical records (EMRs) of 5,098 neonates aged 0–6 months were retrospectively reviewed for the period between March 2019 and March 2022. After meeting the inclusion and exclusion criteria, a total of 4,126 neonates were included in this study. Ethical approval was obtained from the Institutional Review Board at Sidra Medicine (Ref: IRB# 1967270) and the Biomedical Research Ethics Committee at the University of the Western Cape (Ref: BM22/10/23). Given the retrospective design of the study and the use of de-identified data, a waiver of informed consent was granted.

The sample size was calculated using Epi Info 7 (CDC, Atlanta, GA, US), based on two similar retrospective studies.^18,19^ With a 95% confidence interval (CI), 80% power, and an exposure-to-non-exposure ratio of 42:1, the calculation indicated a minimum required sample size of 1,954 neonates.

Universal neonatal hearing screening was conducted using Madsen AccuScreen devices (Natus Inc., Taastrup, Denmark) prior to discharge, with follow-up diagnostic assessments performed by audiologists as indicated.

### 2.2. Inclusion and exclusion criteria

Neonates born and treated between March 2019 and March 2022 who had at least one documented hearing screening during admission were included. Neonates referred for screening but lacking completed tests or with missing screening results were excluded.

### 2.3. Variables

Demographic data included sex, gestational age, birth weight, and nationality. Nationality information was extracted from a de-identified database provided by the institution’s data management team. In cases where nationality was not documented, no additional identifiers were available to retrieve or infer this information. As a result, a proportion of the sample was categorized as “unknown nationality”.

Neonatal risk factors investigated included common JCIH risk factors: hyperbilirubinemia; TORCHS infections (toxoplasmosis, rubella, cytomegalovirus, herpes, syphilis); exposure to ototoxic medications; family history of childhood HL; and meningitis. Additional factors included craniofacial abnormalities (e.g., Chiari malformations, Down syndrome, macro- and microcephaly, microtia with external auditory atresia, cleft lip and palate, and craniosynostosis); lifestyle-related factors (GDM and pre-eclampsia); intrauterine growth restriction (IUGR); and body dysmorphic disorder. Risk factors were identified using International Classification of Diseases, 10th Revision (ICD-10) codes and string searches within the EMR system. Consanguinity was initially considered but was removed due to inconsistent data.

Neonates were categorized as either “well-babies” (no identified risk factors) or “high-risk” (presence of medical complications and comorbidities typically requiring NICU admission for ≥5 days). Initial-stage screening involved OAE testing for well-babies and/or AABR for high-risk neonates after 24 hours as per institutional guidelines, resulting in either a “PASS” (no further testing required) or “REFER” (requiring retesting) outcome based on the infant’s risk status. The screening after 24 hours was intended to reduce the likelihood of false-positive results caused by vernix or residual amniotic fluid in the ear canal or middle ear, which can temporarily impair sound conduction. Careful interpretation is advised, as a proportion of initial “REFER” results may present as false positives.

Second-stage screening was conducted during NICU admission or on an outpatient basis, with results again classified as either “PASS” or “REFER” outcome. Infants who failed two consecutive hearing screenings were referred to the audiology clinic for a diagnostic hearing assessment, which included auditory brainstem response (ABR) testing and tympanometry, when appropriate.

### 2.4. Statistical analysis

The anonymized dataset was checked for errors and compiled in a password-protected Microsoft Excel spreadsheet (Microsoft Corp., Redmond, WA). Statistical analyses were performed using SPSS Statistical Software version 28 (IBM Corp., Armonk, NY). Chi-squared and Fisher’s exact tests were used to examine associations between risk factors and hearing screening outcomes. Logistic regression analyses identified independent predictors of HL, with odds ratios (ORs) reported alongside 95% confidence intervals (95% CIs). Statistical significance was set at p < 0.05 for all analyses.

## 3. RESULTS

### 3.1. Study population characteristics and hearing screening outcomes

**Figure 1:**
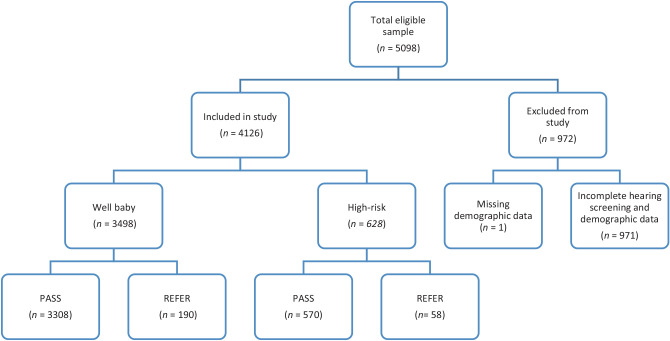
Sample stratification at various stages of data analysis.

Of 5,098 eligible neonates, 4,126 were included in the final analysis. Exclusions were due to incomplete hearing screening data (n = 971) or missing demographic information (n = 1) (Figure 1).

Most neonates were born at the facility: 3,498 were well-babies (84.8%) and 628 high-risk neonates (15.2%). The mean birth weight was 3.14 kg (SD ± 0.53; range 0.5–5.51 kg), with a nearly equal gender distribution (49.4% females, 50.6% males). The most common gestational age was 39 weeks and 1 day. Qatari nationals accounted for 40.0% of the sample, followed by British (3.3%), Indian (2.7%), American (1.8%), and Egyptian (1.5%) nationals (Table 1). Nationality data were missing for 33% of the sample, classified as “unknown”, which may have introduced bias in analyses involving nationality-based comparisons.

Six percent of neonates (*n* = 248) failed the hearing screening, while 94% (*n* = 3,878) passed. The overall screening rate was 80.85%, which is below the JCIH-recommended threshold of 90%.^20^ The test demonstrated a specificity of 93.98% and a sensitivity of 93.99%, slightly below the 95% benchmark.^20^

### 3.2. Bivariate analysis of risk factors

No significant differences were observed between gender and hearing screening results (p = 0.738) (Table 2). Vaginal deliveries were slightly more common (*n* = 2261; 56.6%) than C-sections (*n* = 1735; 43.4%). Again, however, no significant differences were observed between the type of delivery and hearing screening results (*p* = 0.200).

High-risk neonates were more likely to fail the hearing screening than well-baby neonates (*p* < 0.001). Common risk factors, such as GDM (34.5%), hyperbilirubinemia (21.0%), and gentamicin exposure (9.6%), were not directly associated with HL. Interestingly, neonates with hyperbilirubinemia were more likely to pass the hearing screening, with a statistically significant association observed (*p* = 0.034). This finding contrasts with general expectations, as hyperbilirubinemia is usually associated with auditory impairment, yet in this study, it appeared to be linked with a higher likelihood of passing the hearing screening.

### 3.3. Multivariate analysis of risk factors

Neonates with Down syndrome were 14 times more likely to fail the hearing screening (OR = 14.215; 95% CI: 4.286–47.151; *p* < 0.001). Significant associations were also observed in neonates with cleft lip/palate, where these individuals were four times more likely to fail their hearing screening (OR = 4.371; 95% CI: 1.384–13.801; *p* = 0.012); as well as in high-risk (NICU) admissions, where these neonates showed approximately double the odds of failing hearing screening compared to well-baby admissions (OR = 1.973, 95% CI: 1.266–3.076; *p* = 0.003) (Table 3).

Neonates born via C-section were associated with lower odds of failing the hearing screening (OR = 0.753, 95% CI: 0.568–0.999; *p* = 0.049). However, this finding should be interpreted with caution, as the CI is close to the null. Hyperbilirubinemia was also associated with reduced odds of failing the hearing screening (OR = 0.597, 95% CI: 0.408–0.873; *p* = 0.008). Although neonates with Chiari malformation had 4.6 times higher odds of failing the hearing screening (REFER), this association was not statistically significant in the model.

## 4. DISCUSSION

This retrospective study identified significant associations between neonatal risk factors and hearing loss (HL) in Qatar. Notably, Down syndrome and cleft lip/palate were significantly associated with an increased risk of HL. Interestingly, GDM and hyperbilirubinemia were associated with a reduced risk of HL.

### 4.1. Prevalence, sensitivity, and specificity

The referral rate of 6% slightly exceeded the JCIH’s benchmark of 4%.^6^ However, this figure reflects neonates who failed the initial hearing screening; thus, further diagnostic evaluations are needed to confirm HL.

### 4.2. Vernix and transient otitis media

It is important to consider transient otitis media as a potential contributor to failed hearing screening results. Factors such as vernix caseosa in the external ear canal and middle ear effusion related to the birthing process can cause temporary conductive HL (CHL) in the immediate post-natal period.^21,22^ These factors may confound the interpretation of screening outcomes, particularly in retrospective datasets where follow-up diagnostic information may be unavailable. Such factors can result in false-positive screening outcomes, indicating HL where it does not actually exist. There is no universally agreed-upon timeframe for conducting post-natal hearing screening;^23^ however, it is recommended that hearing screening be performed as close to discharge from the facility as possible, with clear follow-up protocols that can be implemented at the primary care level.^24^

### 4.3. Low screening rate

The screening rate of 80.85% fell below the recommended 90% benchmark,^6^ highlighting room for improvement. In Saudi Arabia, the implementation of a national e-system database for hearing screening has successfully increased screening rates from 17% to 89%,^25^ a model that could be adapted for use in Qatar. While the specificity of the program in our study was adequate, its sensitivity was slightly below the 95% benchmark,^6^ suggesting the need for enhanced coverage and detection methods.

Several factors may contribute to the program not meeting international benchmarks, including the lack of comprehensive data management systems, insufficient training and standardization, and issues with public awareness and follow-up mechanisms. High staff turnover and the ongoing need for retraining in the Middle East may also impact program effectiveness. Periodic monitoring and evaluation of the EHDI program, along with robust follow-up protocols, are essential to enhance performance and align with global standards.^24^

### 4.4. Demographics

The predominance of Qatari nationals in our sample is notable, particularly given that nationals constitute only 11% of the total population.^26^ This suggests that our sample reflects a more stable resident population rather than the transient expatriate demographic. Consequently, it provides valuable insights into conditions more prevalent among Arab populations—which are often understudied—while offering a rich dataset that considers the sociocultural background.

### 4.5. Risk factors and hearing loss

Contrary to previous findings,^27,28^ our study found that neonates born to mothers with GDM were less likely to fail hearing screening. Although the result may appear counterintuitive, the literature presents conflicting evidence, with several factors—such as test type and maternal health—potentially influencing outcomes (Table 4). Our study, which included a large sample size, revealed a high prevalence of GDM; however, no statistically significant association with HL was identified. This contrasts sharply with some smaller studies reporting elevated ORs, though these estimates may be inflated due to limited sample sizes and wide CIs.

Carlos-Hiceta and Reyes-Quintos^27^ reported a significant association between neonates born to mothers with GDM and failed hearing screening results. They proposed that GDM may induce metabolic changes affecting the auditory nervous system and the inner hair cells of the cochlea, suggesting a potential link to Auditory Neuropathy Spectrum Disorder (ANSD) rather than purely SNHL. Based on these findings and considering that a large proportion of well-babies in our study were born to mothers with GDM, AABR screening for these neonates may provide better insight into the neural integrity of the auditory system over OAE testing alone.

A systematic review by Aggarwal and Ravi^12^ reported significant rates of failed hearing screening results among neonates but recommended that future studies include larger sample sizes to draw more definitive conclusions. In our study, 34.5% (n = 1,423) of neonates were born to mothers with GDM—a substantially larger sample than those included in the studies reviewed by Aggarwal and Ravi.^12^ Regional variations in GDM management, newborn hearing screening protocols, and population health characteristics may also contribute to the variability observed across these studies. These findings highlight the need for further large-scale research to better elucidate this association.

Similarly, our study found that infants with hyperbilirubinemia were also less likely to fail the hearing screening. Corujo-Santana et al.^33^ examined 796 neonates with hyperbilirubinemia in the Canary Islands and reported a 4.39% prevalence of HL in this population. However, the authors noted that HL in the affected sample could not solely be attributed to hyperbilirubinemia, as approximately half of the affected infants had one or more additional risk factors for HL, most notably exposure to ototoxic medications.^29^

Moreover, the type of screening test employed in this study should also be considered, as emerging research shows that hyperbilirubinemia primarily affects the auditory nervous system,^13,14^ thereby supporting the use of AABR screening, over OAE—which solely provides information on cochlear function. A systematic review by Akinpelu and colleagues^34^ reported that several studies demonstrate a direct correlation between the severity of hearing impairment and elevated serum bilirubin levels. However, the incidence of HL decreased following treatment (e.g. phototherapy or exchange transfusion), suggesting that early identification and timely management may allow for partial or complete reversibility of hearing deficits. In our study, it is plausible that neonates either had serum bilirubin levels below the neurotoxic threshold or received prompt treatment; however, this stratification was not thoroughly investigated. Future research is warranted to further examine the relationship between elevated serum bilirubin levels and auditory outcomes.

The interpretation of the association between hearing screening results and C-section delivery requires further investigation. Although our finding was statistically significant, underlying factors or interactions that could explain this trend should be considered. It is possible that factors such as maternal health, the presence of additional comorbidities, and variations in delivery practices could influence neonatal risk factors not fully captured in our analysis. Additionally, it may be worthwhile to explore potential confounders or sub-group effects that could clarify the observed association between C-section delivery and hearing screening outcomes. Further research with a larger and potentially stratified sample is recommended to better understand these nuances.

### 4.6. Antibiotic exposure

Gentamicin is an aminoglycoside antibiotic commonly administered to treat various bacterial infections, particularly neonatal sepsis, pneumonia, and meningitis.^35^ Although used as a standard first-line antimicrobial agent, its prolonged intravenous administration (for more than 12 hours) is commonly associated with nephrotoxicity and ototoxicity.^35,36^ Despite elevated trough levels (≥2 mg/L) among infants in our cohort, no significant association with HL was observed. This may be attributable to variations in dosing duration or administration frequency. Further studies are needed to clarify this relationship.

### 4.7. Down syndrome and cleft lip/palate

Down syndrome and cleft lip/palate emerged as significant risk factors for HL, displaying varied prevalence rates across previous studies.^37,38^ The association between Down syndrome and HL may involve multiple factors, including middle ear pathologies and atypical craniofacial anatomy, highlighting the need for comprehensive audiological investigations, such as ABR and immittance audiometry.^39^

Similarly, cleft lip/palate, a common craniofacial abnormality, presents significant challenges in speech development and is associated with a higher risk of recurrent ear infections, potentially leading to CHL.^40,41^ Genetic syndromes linked to both Down syndrome and cleft lip/palate can also occur in consanguineous families,^42,43^ highlighting the importance of exploring familial patterns and genetic contributions to HL.

### 4.8. Limitations and strengths

This study relied on retrospective data from a single medical facility, limiting the generalizability of findings to the broader Qatari neonatal population and restricting control over confounding variables. Challenges in obtaining de-identified data—including the absence of standardized recording of consanguinity in patient medical records—also hindered the exploration of its relationship with HL. Investigating consanguinity, reported in approximately 50% of 18 Qatari families^44^ and 83.1% of hearing-impaired children in a Saudi Arabian study,^9^ could provide further insights into HL in the Middle East. Additionally, exclusions due to missing hearing screening data suggest parental awareness and acceptance issues, potentially delaying EHDI processes crucial for early identification of HL in infants.

A key limitation of this study was the lack of nationality data due to reliance on de-identified secondary data. This limited our ability to effectively analyze hearing screening outcomes across specific national or ethnic groups. However, given the relative homogeneity of the neonatal population at the facility and the study’s focus on clinical neonatal risk factors, we believe this limitation is unlikely to substantially affect the main findings.

Investigating the reasons behind parental refusal of hearing screening—such as cultural beliefs, lack of knowledge, or other relevant factors—offers a promising avenue for further research. Additionally, exploring OAE and AABR tests separately—which was not possible in this study due to limited data—could help in understanding different HL types and facilitate collaboration on Qatar-specific neonatal HL data. A further recommendation is to expand this study to include data from multiple facilities across Qatar—both public and private. This approach could enhance the understanding of population-level trends and support the effective and optimal management of EDHI programs nationally.

Some strengths of this study include the large sample size, which enabled a robust epidemiological investigation of neonatal risk factors associated with HL. The final analyzed sample exceeded the required minimum, increasing the statistical power and precision of our findings. This study also offered a unique perspective on the prevalence of context-specific risk factors, which are often underrepresented in neonatal hearing screening programs. To our knowledge, this is the first large-scale epidemiological study in Qatar examining a comprehensive range of risk factors and their association with HL. The findings provide essential local data that can inform national policy and screening guidelines and may also apply to similar international contexts.

## 5. CONCLUSION

The findings of this study highlight the potential value of tailoring neonatal hearing screening programs to locally relevant risk factors. Incorporating conditions such as GDM, pre-eclampsia, and Down syndrome may enhance the early identification of childhood HL in Qatar and similar contexts.

While lifestyle-related conditions such as GDM and pre-eclampsia were not significantly associated with HL, they were prevalent among mothers in the sample population. These findings highlight the need for ongoing public health education to raise awareness of lifestyle-related risk factors for maternal and child health. Clear screening protocols are vital to prevent delayed detection and misidentification of HL in infants. Establishing a national hearing screening database could aid in monitoring and evaluation of the EHDI program in Qatar.

## LIST OF ABBREVIATIONS

**Table:** 

AABR	Automated Auditory Brainstem Response
EHDI	Early Hearing Detection and Intervention
EMR	Electronic Medical Records
GDM	Gestational Diabetes Mellitus
HL	Hearing Loss
JCIH	Joint Committee on Infant Hearing
NICU	Neonatal Intensive Care Unit
OAE	Otoacoustic Emissions
SNHL	Sensorineural Hearing Loss

## DATA AVAILABILITY STATEMENT

The data supporting the findings of this study are available from the corresponding author upon reasonable request.

## ETHICAL APPROVAL

Ethical approval was obtained from the Biomedical Research Ethics Committee at the University of the Western Cape (Ref: BM22/10/23) and Sidra Medicine’s Institutional Review Board (Ref: IRB# 1967270). A waiver of informed consent was granted due to the retrospective nature of the study and the use of de-identified data.

## AUTHORS’ CONTRIBUTION

JA: Conceptualization, methodology, data collection, formal analysis, and writing—original draft. WA and RR: Supervision, formal analysis, writing—review and editing, and ethical oversight.

## FUNDING

This work is supported by Sidra Medicine. All funding is covered under agreements between Sidra Medicine and the respective publishers.

## ACKNOWLEDGMENTS

An acknowledgment is made to Mr. Kirankumar Kandra for his generous assistance with data extraction and compilation.

## COMPETING INTERESTS

The authors hereby declare that they have no conflicts of interest concerning this study.

## Figures and Tables

**Table 1.** Demographic profile of sample neonates (n = 4126) treated at Sidra Medicine from March 2019 to March 2022.

**Variables**	***n***	**%**
**Born at Sidra Medicine**		
Yes	4120	99.9
No	6	0.1
**Neonatal protocol**		
Well-baby	3498	84.8
NICU admitted (total)	628	15.2
**Sex**		
Female	2039	49.4
Male	2087	50.6
**Nationality**		
Qatari	1651	40.0
Unknown*	1347	32.6
British	137	3.3
Indian	113	2.7
American	76	1.8
Egyptian	61	1.5
Other**	741	18.1

*Nationalities included under “Unknown” could be accounted to data capturing, or residence visa issues.

**The nationalities included under the “Other” category comprised minimal percentages of over 80 countries and were too many to include in Table 2.

**Table 2.** Prevalence analysis of characteristics and associated hearing screening outcome.

**Characteristics**	***n* (%)**	**PASS *n* (%)**	**REFER *n* (%)**	*p*-value*
**Total**	4126	3878	248	
Male	2087 (50.6)	1959 (93.9)	128 (6.1)	
Female	2039 (49.4)	1919 (94.1)	120 (5.9)	0.738
**Newborn protocol**				
Well-baby	3498 (84.8)	3308 (94.6)	190 (5.4)	
High-risk (NICU)	628 (15.2)	570 (90.8)	58 (9.2)	<0.001
**Type of delivery**				
C-section	1735 (43.4)	1642 (94.6)	93 (5.4)	
Vaginal	2261 (56.6)	2118 (93.7)	143 (6.3)	0.200
**Risk factors**				
GDM	1423 (34.5)	1344 (94.4)	79 (5.6)	0.368
Hyperbilirubinemia (>100 μmol)	868 (21.0)	829 (95.5)	39 (4.5)	0.034
Gentamicin (>2 mg/L)	397 (9.6)	363 (91.4)	34 (8.6)	0.024
Pre-eclampsia	52 (1.3)	49 (94.2)	3 (5.8)	0.941
IUGR	44 (1.1)	40 (90.9)	4 (9.1)	0.387
Cleft lip/palate	17 (0.4)	12 (70.6)	5 (30.4)	<0.001
Down syndrome	15 (0.4)	8 (53.3)	7 (46.7)	<0.001
Chiari malformation	8 (0.2)	5 (62.5)	3 (37.5)	<0.001
Macrocephaly	5 (0.1)	4 (80.0)	1 (20.0)	0.267^∞^
Craniosynostosis	5 (0.1)	4 (80.0)	1 (20.0)	0.267^∞^
Pierre-Robin association	4 (0.1)	2 (50.0)	2 (50.0)	0.020^∞^
Microtia	3 (0.1)	0 (0.0)	3 (100.0)	<0.001^∞^
Family history of HL	2 (0.0)	1 (50.0)	1 (50.0)	0.117^∞^
Toxoplasmosis	1 (0.0)	0 (0.0)	1 (100.0)	0.060^∞^
Cytomegalovirus	1 (0.0)	0 (0.0)	1 (100.0)	0.060^∞^
Syphilis	1 (0.0)	0 (0.0)	1 (100.0)	0.060^∞^
External auditory canal atresia	1 (0.0)	0 (0.0)	1 (100.0)	0.060^∞^
Body dysmorphic disorder	1 (0.0)	1 (100.0)	0 (0.0)	1.000^∞^

*Chi-squared test for independence.

^∞^Fisher’s exact test (used for less than five observations).

**Table 3.** Multivariate analysis of the most prevalent risk factors associated with hearing loss.

**Risk factors**	**Odds ratio (95% CI) [Unadjusted]**	**Odds ratio (95% CI) [Adjusted]**	***p*-value**
C-Section	0.839 (0.641–1.098)	0.753 (0.568–0.999)	0.049*
High-risk (NICU)	1.772 (1.303–2.409)	1.973 (1.266–3.076)	0.003*
Hyperbilirubinemia	0.686 (0.483–0.974)	0.597 (0.408–0.873)	0.008*
Gentamicin	1.538 (1.055–2.244)	0.885 (0.517–1.513)	0.654
GDM	0.881 (0.669–1.161)	0.913 (0.686–1.215)	0.532
Pre/eclampsia	0.957 (0.296–3.092)	1.088 (0.333–3.559)	0.889
Chiari malformation	9.485 (2.254–39.920)	4.670 (0.884–24.662)	0.070
Down syndrome	14.051 (5.053–39.071)	14.215 (4.286–47.151)	<0.001*
Cleft lip/palate	6.629 (2.317–18.968)	4.371 (1.384–13.801)	0.012*
IUGR	1.573 (0.558–4.432)	0.787 (0.220–2.809)	0.712

*Significant associations with *p* < 0.05.

**Table 4.** Summary of recent studies conducted to determine the association between GDM and hearing loss.

**Authors**	**Context**	***n***	**Prevalence of GDM**	**Prevalence of HL with GDM**	**Odds ratio (95% CI)**
This study	Qatar	4126	34.5%	5.6%	0.913 (0.686–1.215)
Hanege et al.^28^	Turkey	179	42.0%*	0.00%	
Carlos-Hiceta and Reyes-Quintos^27^	Philippines	150	7.0%	40.0%	9.250 (1.95–43.84)
Zhou et al.^29^	China	666	10.36%	4.35%	
Padmadasan et al.^30^	India	120	100%*	4.16%	
Samanth et al.^31^	India	1068	17%	15.8%	
Sharma et al.^32^	India	120	50%*	13.3%	2.153 (0.612–7.580)

*Case–control studies.
